# Practical insights for enhancing primary care rehabilitation services in low-resource contexts

**DOI:** 10.4102/phcfm.v17i1.4699

**Published:** 2025-02-06

**Authors:** Maria Y. Charumbira, Karina Berner, Quinette A. Louw

**Affiliations:** 1 Department of Health and Rehabilitation Sciences, Faculty of Medicine and Health Sciences, Stellenbosch University, Cape Town, South Africa

**Keywords:** rehabilitation, physiotherapy, occupational therapy, speech and language therapy, audiology, primary health care

## Abstract

**Background:**

Rehabilitation services are crucial for addressing the growing burden of functioning problems related to chronic conditions in low and middle-income countries. These services, however, remain limited in South Africa and Zimbabwe’s primary health care (PHC) settings.

**Aim:**

To identify recommendations for enhancing the accessibility and quality of rehabilitation services in PHC in South Africa and Zimbabwe from the perspectives of primary care providers (PCPs) and patients.

**Setting:**

Ten PHC facilities in the Eastern Cape, South Africa and in Manicaland, Zimbabwe.

**Methods:**

A qualitative descriptive study was conducted. Semi-structured interviews were conducted with 37 PCPs and 40 patients. Thematic analysis of transcribed and translated data was done in ATLAS.ti version 22.2.4.

**Results:**

Five key recommendations emerged: (1) provide rehabilitation services closer to home through facility-based services, outreach, home visits and telerehabilitation; (2) improve patient awareness of rehabilitation through patient education, community engagement and patients actively seeking information; (3) enhance PCPs’ knowledge and basic skills in rehabilitation through training and guideline development; (4) improve communication among PHC stakeholders; and (5) advocate for rehabilitation inclusion in leadership, resource allocation and multi-disciplinary teams.

**Conclusion:**

Implementing the identified recommendations could significantly improve the accessibility and quality of rehabilitation services in PHC, aligning with global initiatives to scale up rehabilitation in health systems. Future research should focus on evaluating the implementation and impact of these recommendations.

**Contribution:**

The study emphasises the inclusion of PCPs’ and end-users’ voices in providing practical, context-specific strategies for establishing or strengthening rehabilitation services in low-resource PHC settings.

## Introduction

Globally, a major scaling up of rehabilitation services is needed in response to the current trends in health and ageing.^1^ In low and middle-income countries (LMICs), such as South Africa and Zimbabwe, the growing demand is largely ascribed to populations experiencing longer lifespans, increased non-communicable diseases (NCDs) and injuries, and improved survival rates from previously fatal conditions.^2,3^ Thus, the importance of rehabilitation in addressing the impact of health conditions on individuals’ daily lives, by optimising quality of life and functioning, has become increasingly apparent.^4^

Despite this escalating need, rehabilitation has been a low priority for many countries, especially those with limited health investments. Often considering rehabilitation non-essential, these countries do not allocate specific budgets for this service, which decreases both the availability and the quality of rehabilitation services.^5^ The extent and quality of rehabilitation services vary across population groups and geographical areas according to factors such as urban versus rural settings, availability of foreign funding and supportive national health policies.^6^ Rehabilitation services are provided at selected levels of health systems and are often absent within primary health care (PHC), which serves as the initial contact for most patients seeking healthcare.^7^ For example, only 3% of rehabilitation professionals in South Africa’s public health sector work in PHC,^8^ while in Zimbabwe, they are mostly found at secondary and tertiary levels of healthcare.^9^ Additionally, rehabilitation professionals work mostly in the urban centralised areas or in the private sector.^7^ Consequently, poorer populations must travel long distances and incur increased out-of-pocket expenses to access rehabilitation,^10^ leading to delayed rehabilitation intervention and potentially exacerbating health conditions. The few who can access rehabilitation in PHC often experience underdeveloped and poorly coordinated services.^11^ What is more, poverty, poor health literacy and limited awareness of the primary care patients about the importance of rehabilitation further compound the problem.^10^

Strengthening rehabilitation services within PHC is crucial to address these challenges. While South Africa and Zimbabwe have community-based rehabilitation programmes,^12,13^ these initiatives are often not sustainable because of their dependency on unstable donor funding, which is often targeted at specific diseases.^14^ Ideally, a well-functioning healthcare system should incorporate both PHC-integrated rehabilitation and community-based approaches to ensure comprehensive coverage and accessibility.^15^ Thus, incorporating rehabilitation in PHC in LMICs may enhance equitable access, provide timely identification and management for patients with limited functioning related to chronic health conditions, promote continuity of care and support a holistic, patient-centred healthcare model.^16^ The integration process is ongoing in many LMICs in alignment with the World Health Organization’s (WHO) Rehabilitation 2030 initiative to scale up rehabilitation services at all levels of care to ensure universal health coverage.^16^ However, comprehensive, country-wide integration remains a challenge because of resource constraints and competing health priorities.

To effectively enhance rehabilitation services in PHC, it is essential to obtain valuable insights from those at the forefront of primary care delivery and the end-users of these services.^17^ Patients have the expertise to improve or redesign rehabilitation service delivery through their lived experiences in seeking and utilising healthcare services.^18^ The primary care providers (PCPs) can provide informed views regarding the health system challenges that they face in providing primary care. This collaborative approach ensures that interventions are tailored to local contexts and specific population needs.^17^ By involving stakeholders directly, healthcare systems can develop more sustainable and effective rehabilitation programmes within PHC.

Despite the clear importance of strengthening rehabilitation in PHC, limited information on context-specific strategies has been available in low-resource contexts. A recent scoping review cited the following key enablers to the integration of rehabilitation services in LMICs’ PHC systems: raising awareness of rehabilitation roles, sharing information across different care levels, managing referral pathways and facilitating outreach programmes.^19^ However, practical guidance on how to achieve integration of rehabilitation in PHC, particularly from the perspective of local PCPs and primary care patients, remains limited. Recent studies have explored strategies to integrate specific fields of rehabilitation such as physiotherapy or occupational therapy in low-resource settings.^20,21^ A comprehensive overview of the integration and strengthening of multiple rehabilitation disciplines is required to obtain a holistic perspective. Recommendations from studies conducted in high-income settings may be difficult to implement in countries with limited economic resources, and cultural and political barriers.^22^

This study aimed to provide informed recommendations and context-specific strategies to guide PHC, rehabilitation service planners and policy makers in developing and extending equitable rehabilitation services in PHC.

The study objectives were to: (1) describe the recommendations from the perspectives of PCPs and patients in two low-resource contexts; (2) compare the findings between these two groups; and (3) analyse differences and similarities between the contexts of South Africa and Zimbabwe. The recommendations are intended to promote equitable access to affordable rehabilitation services. They do not provide guidance on clinical interventions.

## Research methods and design

### Study design

A qualitative descriptive design was used to afford the researchers the flexibility to understand human experiences and provide practical recommendations for enhancing rehabilitation in PHC.^23^ This article forms part of a larger doctoral study to understand rehabilitation quality and access in PHC in low-resource contexts. Some study findings have been reported elsewhere.^10,24^ This article focusses on a dominant theme that emerged from the qualitative data analysis and is reported according to the consolidated criteria for reporting qualitative research (COREQ).^25^

### Setting

The study took place in 10 PHC facilities located in two districts of Manicaland, Zimbabwe, and two districts of the Eastern Cape, South Africa. Six of the selected PHC facilities were from South Africa. These provinces are among the poorest in both countries with most of the populations residing in rural areas,^26,27^ and were selected for the significant disparities in healthcare access.

South Africa’s public primary care consists of approximately 3500 clinics and community health centres (CHCs).^28^ Zimbabwe has about 1600 clinics and rural health centres.^29^ In both countries, PHC systems are predominantly nurse-led, with medical officers staffed in some PHC facilities.^30^ Provision of PHC facility-based rehabilitation services in both settings is largely dependent on the district health system through outreach and home visit programmes. South Africa provides an example of successful (albeit suboptimal) integrated care models at primary level, with larger PHC facilities offering daily outpatient rehabilitation services.^11^ However, in Zimbabwe, rehabilitation professionals are not typically found in PHC facilities.^9^

### Sampling and recruitment of participants

Patient participants were purposively sampled to ensure diverse demographic characteristics (e.g. age, gender, location) and varying rehabilitation experiences. Inclusion criteria for participants were: (1) adults (18 years or older) or their carers; (2) any health-related functioning problems as described by the International Classification of Functioning Disability and Health Framework (ICF)^31^; and (3) willingness to participate. Exclusion criteria included inability to communicate because of cognitive or speech impairments without a carer to provide their perspective. The first author recruited participants from the PHC facility waiting rooms. The primary care nurses introduced the research team to the patients and provided opportunity to explain the study purpose. Interested patients completed a brief demographic questionnaire. A quick analysis of the participants’ response enabled the first author to identify eligible participants. Thereafter, criterion-based sampling was used to select participants with varied demographic characteristics and medical history and obtain written informed consent.

The PCPs were purposively sampled based on the following inclusion criteria: (1) providing primary care to adult patients; (2) at least six months experience in PHC; and (3) willingness to participate. A maximum variation approach was used to select for diversity in age, gender, healthcare profession, location and years of PHC experience. Student PCPs were excluded. Recruitment included snowball sampling through in-person communications and e-mails. Prospective participants completed a short survey to confirm eligibility, followed by written informed consent. Data collection was conducted concurrently with the data analysis, which allowed for ending the recruitment processes once data saturation was achieved, that is, when adequate information had been obtained to address study objectives and no new themes arose from subsequent interviews.^32^

#### Description of sample

A total of 37 PCPs were interviewed – 22 from South Africa and 15 from Zimbabwe. The PCP participants consisted of 30 non-rehabilitation PCPs and 7 rehabilitation professionals (6 from district and 1 from PHC). The PCPs’ median age was 42 years and ranged from 27 to 63 years (More detailed descriptions of this sample have been published elsewhere).^24^

In all, 40 primary care patients were interviewed. Their demographics and other characteristics are presented in Table 1. Most patients experienced pain-, mobility- and mental-related functioning problems.

**TABLE 1:** Sociodemographic and clinical characteristics of participants.

Variable	South Africa (*n* = 25)					Zimbabwe (*n* = 18)					Total (*N* = 43)				
	Mean	s.d.	Range	*n*	%	Mean	s.d.	Range	*n*	%	Mean	s.d.	Range	*N*	%
Age (years)	52.4	14.84	20–74	-	-	52.1	16.28	25–80	-	-	-	-	20–80	-	-
Married	-	-	-	10	40	-	-	-	13	72	-	-	-	23	53
**Education**															
None	-	-	-	3	12	-	-	-	1	6	-	-	-	4	9
Primary school	-	-	-	11	44	-	-	-	3	17	-	-	-	14	33
Secondary school	-	-	-	9	36	-	-	-	12	67	-	-	-	21	49
Tertiary	-	-	-	2	8	-	-	-	2	11	-	-	-	4	9
Employed	-	-	-	6	24	-	-	-	4	22	-	-	-	10	23
**Source of income**															
Vending farm produce or second-hand clothes	-	-	-	0	9	-	-	-	50	9	-	-	-	21	-
Pension	-	-	-	7	28	-	-	-	3	17	-	-	-	10	23
Salary or wages	-	-	-	6	24	-	-	-	3	17	-	-	-	9	21
Government grant	-	-	-	10	40	-	-	-	0	10	-	-	-	23	-
None (supported by family)	-	-	-	2	8	-	-	-	3	17	-	-	-	5	12
**Chronic health conditions**															
More than one chronic health condition	-	-	-	-	76	-	-	-	-	72	-	-	-	-	72
Top condition: % living with HIV (% on ART)	-	-	-	56	96	-	-	-	56	100	-	-	-	56	96
Used assistive devices (wheelchairs, crutches, walking sticks)	-	-	-	-	32	-	-	-	-	17	-	-	-	-	26
**Received rehabilitation at primary care**															
Yes	-	-	-	-	12	-	-	-	-	0	-	-	-	-	7
No	-	-	-	-	88	-	-	-	-	100	-	-	-	-	93

ART, antiretroviral therapy; HIV, human immunodeficiency virus; s.d., standard deviation.

#### Data collection

Patient participants were interviewed face-to-face in PHC consultation rooms, while PCP participants were given the choice of in-person, phone or video-conference interviews at their convenience. Each interview lasted 20 min – 30 min and was audio-recorded. To encourage participants to provide honest responses, non-participants were not permitted to enter the rooms during the interviews. The semi-structured interviews were conducted in the participants’ preferred language (English, Shona or isiXhosa) following a scheduled guide that was developed from similar studies on improving integrated PHC.^33,34^ The interview guides^10,24^ included open-ended questions and follow-up probes, which enabled interviewers’ flexibility to elicit detailed information and clarification of concepts raised by the participants. Patient questions focussed on their needs, expectations and preferences for rehabilitation care (whether received or not). The patients were further asked how their experiences could be improved. Primary care providers were asked how rehabilitation services could be better provided and how primary care patients’ access to the services could be enhanced. The interview guides were piloted among non-medical researchers who understood the local languages. The first author directed the English and Shona interviews, while the research assistant took comprehensive notes (both are female physiotherapists with qualitative research experience). A professional medical translator helped conduct the isiXhosa interviews. The first author took field notes during and after each interview recording non-verbal cues and her own reflections. No repeat interviews were required.

#### Data analysis

Transcripts were professionally transcribed and checked against recordings for accuracy by the first author. The Shona and isiXhosa recordings were translated into English by the translation service and verified by bilingual research assistants. Transcripts were sent back to consenting participants for feedback on data accuracy in reflecting their responses. Using ATLAS.ti version 22.2.4, the first author performed inductive thematic analysis following the six-step iterative process described by Braun and Clarke^35^: (1) familiarisation with data through repeated reading of data; (2) generating initial codes by highlighting sections of text and assigning labels; (3) searching for themes by grouping initial codes; (4) reviewing and refining themes; (5) defining and naming themes; and (6) producing the report that presents the themes; with supportive quotes to tell a story from the data that addresses the research questions. Emerging themes and codes were agreed jointly in parallel discussions with the research team, resolving any disagreements through consensus building. Thus, a code-book was generated, which the first author used to code all transcripts.

#### Reflexivity

The first author conducted a reflexive analysis,^23^ acknowledging any assumptions, beliefs or personal biases that may have affected the research process. Her previous 9 year-clinical experience in low-resource contexts may have influenced her understanding of the studied contexts and interpretation of participants’ responses. Continual reflections, thoughts, actions and decisions were documented throughout the research process in a reflexive journal. Engagement with the wider research team in dialogue ensured transparency and critical examination of the research process.

#### Trustworthiness

To ensure credibility (truth value), triangulation was done using multiple data sources, methods or researchers to cross-verify the findings. Confirmability was achieved through member checking by sharing the findings with PCP participants to validate the accuracy of the data interpretation. There were difficulties in sharing findings with patient participants because of loss of contact, language barriers without a professional translator and the lack of electronic devices. However, confirmability was further ensured through peer debriefing and reflexive journaling. Dependability was established by maintaining a clear audit trail involving comprehensive documentation of methodology and decisions made during the study. In terms of transferability, detailed descriptions of the data collection, analysis and interpretation were provided.

#### Ethical considerations

All procedures performed in studies involving human participants were in accordance with the ethical standards of the institutional and/or national research committee and with the 1964 Helsinki Declaration and its later amendments or comparable ethical standards. The study (project research number: 19376) was approved by the Stellenbosch University Health Research Ethics Committee (S21/01/002 [PhD]) and Medical Research Council of Zimbabwe (MRCZ/A/2916). The Permanent Secretary to the Ministry of Health, Provincial Medical Directorate of Manicaland Province, District Medical Officers and PHC facility managers granted study permission in Zimbabwe. The Eastern Cape Department of Health, District Offices of the Department of Health and PHC facility managers granted study permission in South Africa. Prior to each interview, voluntary written informed consent was obtained from all study participants for participation and publication of anonymised data. All data were anonymised by removing any names from the transcripts. Electronic recordings and identifying information were kept in an access-restricted location.

## Results

**FIGURE 1:**
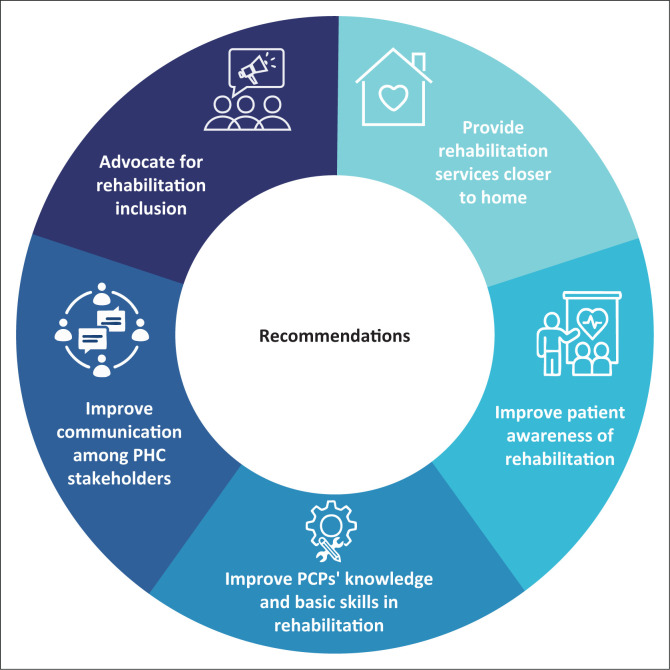
Five major themes or major recommendations identified from the study.

Five major themes representing the major recommendations were identified from the analysis of the participants’ responses (Figure 1).

Each major recommendation included sub-themes or sub-recommendations, which are described next along with supportive quotes. Each quote is labelled with the participant’s code (‘PCP’ indicating non-rehabilitation PCP; ‘P’ indicating a primary care patient and ‘RP’ indicating rehabilitation professionals, location [urban or rural] and the country).

### Recommendation 1: Provide rehabilitation services closer to home

**FIGURE 2:**
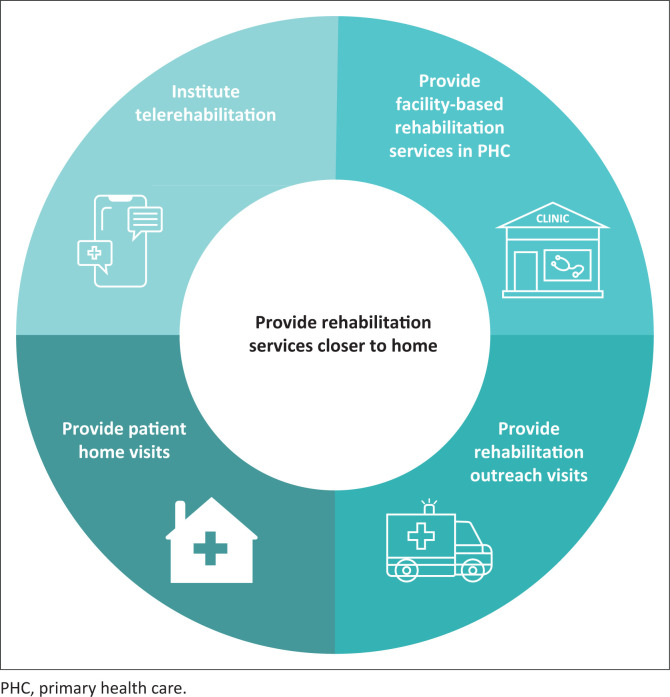
Theme 1 and four sub-themes related to the provision of rehabilitation services closer to primary care patients’ homes.

The first recommendation related to bringing rehabilitation services closer to primary care patients’ homes (Figure 2). Four sub-themes highlighted the methods that could be used to achieve this recommendation.

#### Provide facility-based rehabilitation services in primary health care

Patients expressed the need for facility-based rehabilitation services to be available at local clinics rather than centralised facilities. They highlighted the logistical and financial challenges of accessing distant services at higher-level healthcare institutions:

‘It was always our wish that the clinic must have rehab … Because if you want to go to [*District hospital*], you need to hire a car. Then we go there and for us to return home, we must hire again. It’s expensive for us.’ (P13, RURAL, ZW)

‘If these services come closer to us, we are better served … we can walk to the clinic and in an event that I cannot walk, I might need a small amount for people to push me in a wheelbarrow or hire a scotch cart.’ (P15, RURAL, ZW)

Walking appeared to be the primary means of transportation for many individuals in these contexts. This suggests that rehabilitation services located within walking distance could be more accessible to the community. To ensure effective rehabilitation, services must be easily reachable, taking into account patients’ mobility and financial constraints.

#### Provide rehabilitation outreach visits

Patients recognised that it may not be possible to have continuous rehabilitation services at each clinic. They suggested that rotational rehabilitation outreach services be implemented. One participant indicated a realistic and potentially manageable frequency for such services, considering resource constraints:

‘Isn’t it possible for the rehabilitation facilities to rotate in PHCs … so that those without the means to access hospitals may also be assisted? At least if they can visit here once a year, it would be better … just once a year. If they come and tell me what to do with my back that’s painful, that can really help … while they are here with us.’ (P10, RURAL, ZW)

Patients valued that personal engagement with rehabilitation professionals, although infrequent, would provide a critical touchpoint for them to receive professional guidance and that contact could significantly impact their well-being.

The PCPs concurred with patients regarding the need for regular outreach visits, but further highlighted the importance of logistical planning and communication with rehabilitation professionals. Additionally, outreach efforts to inform the community about these services would be crucial:

‘If there’s some outreach programs, like you visit us once a month, you tell us in advance that you’ll be coming to the facility … then we mobilise our clients who may need those services.’ (PCP9, RURAL, ZW)

Noteworthy is how the PCPs realised the importance of this engagement to be more frequent, suggesting monthly visits as compared to patients who requested annual visits.

The patients suggested that rehabilitation professionals conduct specialised clinics during their outreach visits that focus on the prevalent health conditions within the community. The approach was thought to effectively utilise healthcare resources while providing the required engagement with rehabilitation professionals:

‘Maybe you can come to the clinic … then you indicate the specific type of conditions that you want to assist people with. You then invite the people and help them. That’s the type of help that I think would be helpful … But the issue is that the kind of assistance that we would need from you is through direct engagement as we are doing right now … Because there are so many of us in this area who need assistance.’ (P12, RURAL, ZW)

#### Provide patient home visits

The PCPs viewed home visits as opportunities for collaboration with rehabilitation professionals, building their capacity to guide and monitor patients independently when rehabilitation professionals were unavailable. They valued this approach’s potential to bridge the rehabilitation service gap and ensure continuity of care:

‘We don’t have outreach services for rehab but maybe we can engage with them. When we want to do home visits, we can go together with the rehab tech [*rehabilitation technician*] … we assist the patient together whilst the rehab tech is teaching the patient how to exercise. Then later during the absence of the rehab, when we go alone as community health nurses, we can monitor what they are doing.’ (PCP1, URBAN, ZW)

The patients expressed willingness to receive home visits for rehabilitation services. They indicated trust in the rehabilitation professionals being able to provide a beneficial and convenient service:

‘Of course, I would. If they just visit my house and tell me that they want to help me, I will accept their help.’ (P11, RURAL, ZW)

#### Institute telerehabilitation

Notably, patients recommended the use of digital and mobile communication technologies to provide healthcare. None of the PCPs made this recommendation:

‘Something that I wish can be done is to have communication with the clinic. Maybe over the phone.’ (P7, URBAN, SA)

### Recommendation 2: Improve patient awareness of rehabilitation

Patients realised that obtaining knowledge and information about rehabilitation and self-management strategies was essential. Rehabilitation awareness enhanced their autonomy in healthcare and reduced unnecessary medical interventions. It also empowered them to address functioning problems in the absence of rehabilitation professionals:

‘… many of us lack enough knowledge about rehabilitation issues. This would certainly prevent us from going to the doctors too much. You know the doctors will always have something to say and at times we end up paying a lot of money for the prescribed medicines, yet we could have assisted ourselves through such exercises.’ (P4, URBAN, ZW)

**FIGURE 3:**
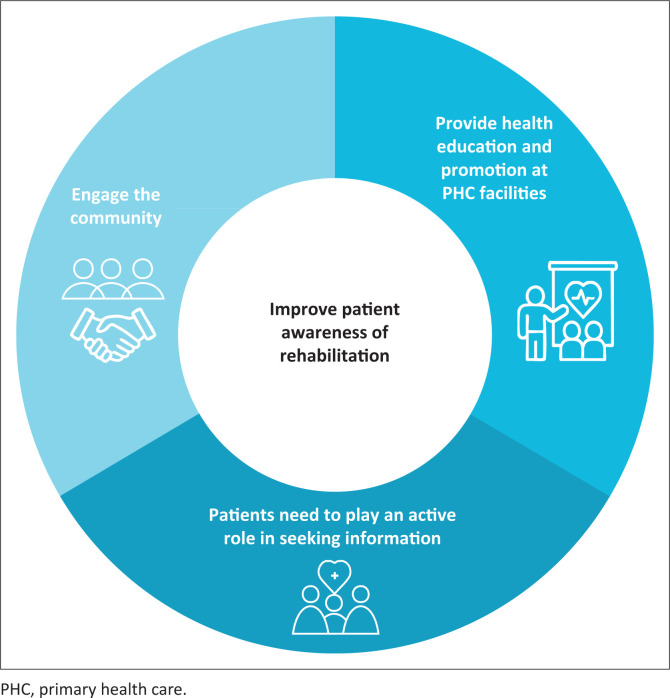
Theme 2 and three sub-themes related to improving patients’ awareness of rehabilitation.

Three sub-themes highlighted how the primary care patients’ awareness of rehabilitation could be increased (Figure 3).

#### Provide health education and promotion at primary health care facilities

The potential strategies for increasing rehabilitation awareness suggested by the patients included providing health education talks and materials at the clinics. The PCPs, particularly the nurses, were mostly expected to be the providers of patient education:

‘You can advertise the service and paste them in clinics in such spaces [*noticeboards*].’ (P4, URBAN, ZW)

‘I think the nurses should come and educate the people during health education sessions or when people come to seek medical help.’ (P10, RURAL, ZW)

The PCPs suggested similar strategies for increasing patients’ awareness, including designing and distributing educational material such as flyers, pamphlets and charts, and conducting health education talks. The nurses realised that even though they were providing the latter at the clinics, they needed to include rehabilitation topics:

‘… health education to our patients during the time they are still waiting in waiting area, yes we do that but our … focus is only about those patients who are on ART … so I think we need to educate more about all things like rehabilitation.’ (PCP15, RURAL, ZW)

Although the patients expected the nurses to primarily provide the information, the nurses recommended that the rehabilitation professionals conduct the educational talks. The novelty of varied professions was thought to potentially renew the patients’ interest, thus enhancing the educational efforts:

‘Because some clients they are used to us. I’ve been here for 16 years [*laughs*] … So, if they see a new face, they are telling them today I want to educate you about rehabilitation, they pay more attention.’ (PCP9, RURAL, ZW)

#### Patients need to play an active role in seeking information

Patients recognised the need to play their part for them to benefit from patient education, by reporting their functioning problems to the PCPs and being proactive in seeking information regarding rehabilitation services:

‘I think, if somebody visits the clinic, they should tell the nurses the truth, then the nurses can educate them accordingly.’ (P3, URBAN, ZW)

‘I guess, for them to explain, one should have gone to look for them.’ (P18, RURAL, ZW)

#### Engage the community

A key recommendation for increasing awareness of rehabilitation involves engaging the community. Both the patients and PCPs suggested that the rehabilitation professionals conduct awareness campaigns at community gatherings:

‘… or even to do awareness campaigns where people are educated whenever they gather, even at funerals or village health workers should also be taught the importance of rehabilitation.’ (P10, RURAL, ZW)

‘Do outreach educations where you do talks in schools, public talks like visit especially informal settlements.’ (PCP14, URBAN, ZW)

Patients from both South Africa and Zimbabwe highlighted how the PCPs could leverage the close-knit nature of the community to disseminate appropriate knowledge regarding rehabilitation:

‘Because if I receive any information, I should share it with whoever cares to come and check on me. I would say, in this community, I would want to see my friend receiving the same help. I also go and check on them if they are bedridden … They said I should do this and that – and we assist each other. I won’t lie to you – that is how we operate here.’ (P12, RURAL, ZW)

‘… people will know that … there will be physiotherapists there and anyone who has problems related to that will be assisted. Just like me, I will also spread the word. (P10, RURAL, SA)

In the same vein, PCPs cited how involving community leaders could improve the effectiveness of community awareness campaigns.

‘For things to be accepted in rural areas mostly, we need village heads. So, if the information first reaches the village head it would be easily accepted in the rural areas.’ (PCP2, RURAL, SA)

### Recommendation 3: Improve primary care providers’ knowledge and basic skills in rehabilitation

**FIGURE 4:**
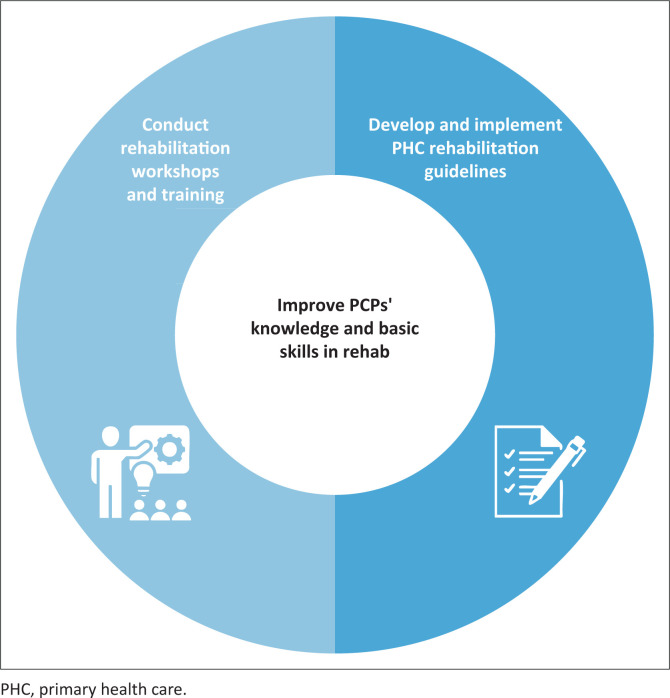
Theme 3 and two sub-themes related to improving primary care providers’ knowledge and basic skills in rehabilitation.

Two sub-themes were identified regarding how to increase PCPs’ knowledge of rehabilitation (Figure 4).

#### Conduct rehabilitation workshops and training

The PCPs called for regular workshops and training sessions to empower them with knowledge and skills for basic rehabilitation. The PCPs needed to understand the rehabilitation needs of the primary care patients they consulted and know which patients to refer to rehabilitation:

‘I think there is a need for conducting some workshops … So that we can be taught on how best we can help our clients and … I know also how we can handle some certain rehab cases at our clinics … As nurses we are very flexible. Like at the clinic, we do most of the work so, if we can do a short course, it would be good for us to help the minor cases then we refer severe cases.’ (PCP1, URBAN, ZW)

#### Develop and implement primary care rehabilitation guidelines

Primary care providers highlighted the need for standard practical guidelines on identification, referral and provision of basic rehabilitation services in PHC to improve their competence. Well-defined roles related to rehabilitation were thought to facilitate better collaboration among the multidisciplinary team members:

‘If we’ve got some job descriptions, if we’ve got some guidelines … for everyone and then all health staff gets to know what we can do with our patients on rehabilitation.’ (PCP6, RURAL, ZW)

‘… the manual or guidelines … so if we are taught then you really know how to handle the client, even the things you are using on the client.’ (PCP7, RURAL, ZW)

### Recommendation 4: Improve communication among primary health care stakeholders

**FIGURE 5:**
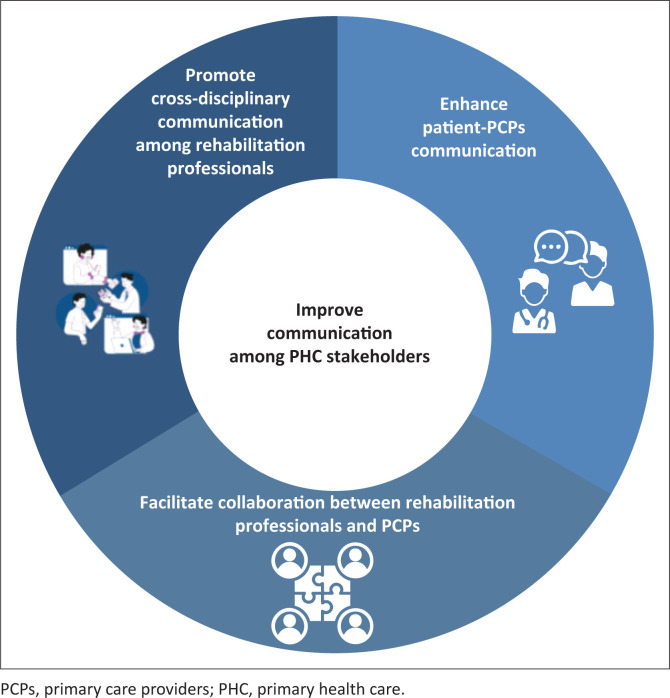
Theme 4 and three sub-themes related to improving communication among primary health care stakeholders.

Three sub-themes were identified indicating how communication could be improved among the various PHC stakeholders (including patients, PCPs and rehabilitation professionals) (Figure 5).

#### Enhance patients-primary care provider communication

Patients recognised that two-way communication between themselves and their PCPs was crucial to receiving optimal healthcare. The patients suggested having anonymous platforms through which they could voice their concerns without feeling intimidated:

‘I believe that there should be things like suggestion boxes in every clinic … Sometimes it is not easy to tell the nurse or to ask for such information because you feel like you are telling them what to do or teaching them their job.’ (P5, URBAN, SA)

In South African contexts, PCPs recommended feasible strategies to overcome the language barriers that were present between rehabilitation professionals and patients such as the use of translators (professional or family):

‘… ask them to bring someone that they trust to come and translate so that we can relay the information we have.’ (P14, URBAN, SA)

#### Facilitate collaboration between rehabilitation professionals and primary care providers

The PCPs appreciated the need to facilitate regular meetings between rehabilitation professionals and clinic staff to foster collaboration:

‘We need to hold some meetings with the rehab team … then we discuss some challenges and the way forward for the clients.’ (PCP1, URBAN, ZW)

‘We need to improve the teamwork, to coordinate and communicate effectively …’ (PCP4, URBAN, ZW)

#### Promote cross-disciplinary communication among rehabilitation professionals

In South Africa, even though rehabilitation was offered at the primary care level, the absence of some rehabilitation disciplines meant that better communication was needed to provide basic rehabilitation, for example, speech therapy in the absence of a speech therapist. Telerehabilitation was suggested as a means of improving communication between rehabilitation professionals:

‘… having a speech therapist in your district that you know you can phone and get some telephonic advice …’ (RP22, RURAL, SA)

### Recommendation 5: Advocate for rehabilitation inclusion

**FIGURE 6:**
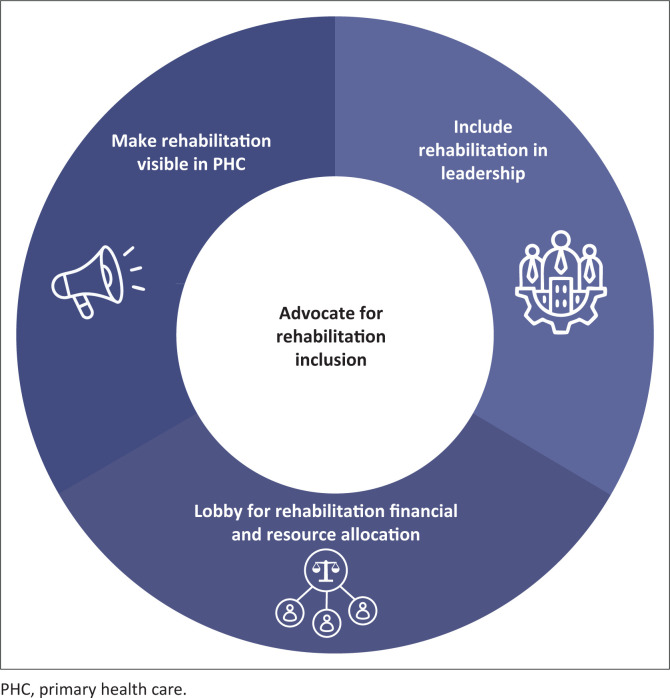
Theme 5 and three sub-themes related to advocating for rehabilitation inclusion at local and national levels.

Figure 6 shows the three sub-themes related to advocating for rehabilitation inclusion in leadership roles, resource allocation and policy at the local and national levels.

#### Include rehabilitation in leadership

The rehabilitation professionals advocated for the inclusion of rehabilitation professionals in leadership roles at facility level:

‘I think the rehabilitation workers need to be included in …management.’ (RP6, URBAN, SA)

#### Lobby for rehabilitation financial and resource allocation

Both PCPs and patients recognised the need for the allocation of finance and resources to support rehabilitation (transport, space and equipment):

‘Maybe if transport services will be provided that is just waiting for the rehabilitation centre …’ (PCP7, RURAL, ZW)

‘Like for this wheelchair which we are using, which is broken, if you could get help even from donors to get a better wheelchair, so that … we can carry him to the clinic … wheelchairs are expensive to buy. We can’t.’ (P13, RURAL, SA)

Patients were also wary of the need for financial investment to equip PHC systems with the necessary infrastructure, diagnostic equipment and essential medicines. They recognised that if rehabilitation was to be nested within PHC, the gaps in PHC systems needed to be addressed first:

‘The health professionals and Government must ensure that we receive ample help and enough medication here in the rural areas. They must also provide us with X-rays so that we can be examined if we have such problems as back pain … so that they can check the extent of damage because right now we are just being given tablets for killing the pain.’ (P15, RURAL, ZW)

One patient who had experienced relatively better PHC in a neighbouring country commented:

‘Things are not of the right standard. If things were at a standard that we witnessed in South Africa, Zimbabwe would never have problems around people’s health. In South Africa, they have all the resources – they might fail to fully utilise them all, but they have them.’ (P9, RURAL, ZW)

#### Make rehabilitation visible in primary health care

Advocacy by rehabilitation professionals was recognised as essential to increasing the visibility and recognition of the rehabilitation profession within the multidisciplinary team:

‘… there are so many people with so many challenges which we meet but those people they never get help simply because the rehabilitation seems far away.’ (P4, URBAN, ZW)

## Discussion

This study sought to understand the recommendations from adult primary care patients and providers to enhance the accessibility and quality of rehabilitation services at this level of healthcare in low-resource contexts. Five key recommendations were identified that require interventions addressing both the supply and demand for rehabilitation services among adult primary care patients.

The patients, PCPs and rehabilitation professionals were unanimous in recommending that rehabilitation services be in closer proximity to the people. Previous studies have highlighted the significant barriers faced by individuals when accessing rehabilitation services, including unavailability of transport, long travel distances and associated costs.^36^ Rural remote areas are doubly disadvantaged because of rough terrain, poor road infrastructure, non-existent public transport and few individuals owning personal vehicles.^36^ Both South Africa and Zimbabwe have implemented PHC policies advocating for health services to be within a 5 km radius of communities, as outlined in South Africa’s National Health Insurance White Paper (2017) and Zimbabwe’s National Health Strategy (2021–2025).^29,37^ This goal reflects a commitment to equitable healthcare access. Integrating rehabilitation services into accessible PHC clinics aligns with the WHO’s framework for integrated people-centred health services.^38^ This approach would help extend the reach of this specialised service, potentially reducing the burden on secondary and tertiary hospitals.^38^ It would also mitigate the prohibitive costs that patients in these settings would otherwise incur travelling to distant healthcare institutions. Additionally, early intervention and management of disabilities and functioning problems (i.e. impairments of body function and structure, activity limitations and participation restrictions)^31^ may ultimately enhance the quality of life for individuals requiring rehabilitation services.^36^ The current study highlighted four methods of providing rehabilitation services at the PHC facilities: facility-based rehabilitation, outreach, home visits and telerehabilitation. The suggested methods indicate that the primary care patients and providers value the presence of rehabilitation professionals at this level of care, even if only briefly. This additional perspective strengthens the case for PHC policymakers and service planners to establish rehabilitation services where they are currently absent and enhance existing primary care rehabilitation services.

Regarding facility-based rehabilitation, significant planning and financial investment would be required for its establishment. This investment includes the recruitment of diverse rehabilitation professionals (e.g. physiotherapists, occupational therapists, speech and language therapists, audiologists) in PHC.^39^ According to the World Health Report, countries with less than 2300 health professionals per million people typically struggle to provide sufficient coverage for PHC interventions and are unable to offer adequate rehabilitation services.^40^ Most LMICs reportedly have population-adjusted ratios of less than one rehabilitation professional per 10 000 capita.^41^ This shortage of rehabilitation professionals necessitates the training of more rehabilitation professionals and provision of incentives that attract and retain rehabilitation professionals in remote or underserved areas.^42^ These incentives include educational opportunities, improved living conditions and financial allowances.^42^ For example, in South Africa, newly graduated therapists required to serve one year in rural remote areas are provided rural health allowance.^43^ Thus, the community service therapists constitute about a third of rehabilitation workforce in South Africa’s PHC.^8^ However, these positions are contingent and may not be filled again once the community therapists complete their term. This lack of permanent posts for community service therapists impacts the long-term availability of rehabilitation services in rural and underserved areas.

Further investment in establishing facility-based rehabilitation in PHC would require providing adequate infrastructure and equipment to enable the newly established rehabilitation professionals to provide optimal care in these settings. Considering the settings’ resource constraints, it would be critical to prioritise what is most concerning to the defined communities.

In the absence of rehabilitation professionals, the remaining suggested methods (outreach, home visits and telerehabilitation) may be cost-effective ways of extending rehabilitation services without the need for permanent staffing at every clinic. A recent South African study demonstrated telerehabilitation as a viable alternative to traditional facility-based rehabilitation during coronavirus disease 2019 (COVID-19) pandemic, which overcomes barriers such as distance and reduces financial burden.^44^ The rehabilitation professionals can use synchronous (video calls) and asynchronous (pre-recorded videos and WhatsApp groups) methods to remotely assess and guide patients through their rehabilitation programmes, limiting the need for in-person visits.^44^ In low-resource settings, there may be a need to consider barriers such as poor connectivity, electricity outages and inadequate technology.^45^ Meanwhile, outreach and home visits seem feasible methods currently employed in the studied settings.^46^ Proper funding and logistical planning would be essential to ensure the success and sustainability of such programmes. This supportive infrastructure includes transportation for professionals, equipment and scheduling.^11^ As mentioned by the patient participants in our study, the outreach programmes need to target the unique needs of the communities. Thus, regular community rehabilitation needs assessments may help rehabilitation professionals identify gaps in care and tailor their services accordingly to avoid wasting resources on care that is not demanded.

The current study underscored the potential role that existing PCPs could play in enhancing or integrating rehabilitation within PHC. The findings emphasised the need for PCPs to better understand the patients’ functioning problems, actively identify patients with rehabilitation needs during routine consultations and refer these patients timeously to rehabilitation. Gilmore et al. reported how such practices are necessary to avoid delayed, inappropriate or inadequate rehabilitation referrals.^47^ Notably, the current study highlighted non-rehabilitation PCPs’ willingness to take on the added role of providing basic rehabilitation services in the absence of rehabilitation professionals. Similarly, rehabilitation professionals wanted to learn from other rehabilitation disciplines to provide basic rehabilitation. For example, a physiotherapist would acquire basic speech therapy skills to assist patients in the absence of a speech and language therapist (SLT). This readiness persisted despite the well-documented challenges of work overload and time constraints reported in these settings.^48^ This attitude may stem from their recognition of rehabilitation’s value at the primary care level in preventing secondary complications of chronic conditions, unnecessary hospitalisation and readmissions.^49^ Furthermore, this willingness to expand their roles aligns with the concept of task-shifting in low-resource settings.^50^ However, implementing such strategies requires careful consideration of training needs and support systems.^50^ Non-rehabilitation PCPs may lack the depth of training needed to deliver certain rehabilitation interventions safely and effectively, potentially compromising patient care. Additional ethical concerns arise regarding role boundaries and expectations placed on already overburdened PCPs to take on additional duties.^50^ For instance, task-shifting without adequate support or compensation might affect their fulfilment of primary responsibilities or contribute to burnout. Recommendations from our study include providing specialised training and workshops, developing primary care rehabilitation guidelines and facilitating teleconsultations with rehabilitation professionals to provide ongoing mentorship and training. An important implication arising from this recommendation will be the need to develop training resources and tools for building PCPs’ basic rehabilitation competencies.

Parallel to the provision of rehabilitation services, the current study emphasised the critical need to increase awareness of rehabilitation among patients and the communities. For patients, recommendations include implementing targeted patient education during consultations, distributing rehabilitation-related educational materials and conducting health promotion talks on rehabilitation topics in clinic waiting rooms. These strategies align with previous research that demonstrated the effectiveness of similar educational interventions in empowering patients in their healthcare interactions and self-management.^51^ Recent work on patient education in South African rehabilitation settings emphasised that patient education materials should be culturally appropriate and ensure language accessibility.^52^ Additionally, the current study suggests engaging the broader community through awareness campaigns in schools and community gatherings such as funerals, with a particular emphasis on involving community leaders to enhance impact. An approach that is deliberately and sensitively inclusive of existing community structures is supported by O’Mara-Eves et al., who found that community-based health interventions, including education, advice and skills development training, addressed health inequalities in disadvantaged communities.^53^ By addressing awareness and knowledge gaps about rehabilitation among patients and their communities, these recommendations have the potential to significantly enhance PHC patients’ rehabilitation service utilisation.

In this study, effective communication across stakeholders at multiple levels of the health system and other sectors was recommended as crucial for enhancing or integrating rehabilitation services in PHC. At the clinical level, poor interdisciplinary collaboration leads to inadequate, inappropriate or delayed rehabilitation referrals resulting in fragmented care and suboptimal health outcomes.^43^ Strategies such as regular multidisciplinary team meetings, joint visits and shared electronic health records can enhance information exchange and care continuity.^54^

Given the reported poor communication with patients in these settings, PCPs and rehabilitation professionals require training in this domain, including patient-PCP cooperation, refining referral routines and enhancing the flow of patient information among healthcare providers and other sectors.^10^ At the community level, engaging community health workers as liaisons has been found to enhance understanding of rehabilitation needs.^55^ Additionally, creating platforms for dialogue between policymakers, healthcare providers and community representatives can ensure context-specific policies and align workforce expansion and financing structures with local needs and resources.^56^ These broader communication efforts support advocacy for rehabilitation inclusion in PHC leadership and governance at local and national levels. The current study identified initial steps, which include making the current leadership and multidisciplinary team aware of the importance of rehabilitation. Increasing the profession’s visibility and recognition may involve interprofessional education, grassroot mobilisation through public awareness campaigns on various media platforms and presenting a case to targeted policymakers.^57^ The latter may require building evidence demonstrating the economic and outcome-related value of rehabilitation. This multi-level communication-driven approach is key to overcoming the systemic barriers that have historically limited the establishment and development of rehabilitation services in PHC in these low-resource contexts.

Differences in country settings were closely related to the performance statuses of the PHC systems. Thus, recommendations from Zimbabwe predominantly focussed on establishing rehabilitation services in PHC rather than enhancing existing primary care rehabilitation services. Currently, the core service delivery functions of PHC in Zimbabwe are severely suboptimal, with inadequate medicines, primary care workforce and infrastructure. More public spending on PHC will be required to strengthen the country’s overall PHC framework before rehabilitation can be nested within it.^58^ While South Africa also experiences inequities in health service provision, with the rural and high-density urban locations experiencing challenges that are very similar to Zimbabwe, it had some rehabilitation services in PHC. This difference is also reflected in the existence of recent research on strengthening rehabilitation within South Africa’s PHC settings, while there is a significant dearth in Zimbabwe. Because financial constraints were not the only barrier to accessing quality rehabilitation services, several recommendations from our study, including increasing awareness and task-shifting, demonstrate the potential for innovative low-cost strategies in enhancing primary care patients’ access to existing rehabilitation services while moving towards establishing primary care rehabilitation. However, while some differences were observed between South Africa and Zimbabwe, many of the core recommendations applied to both contexts. This commonality suggests that the findings may have broader relevance to other low-resource contexts facing similar challenges in rehabilitation service provision.

### Study limitations

Although this study provided recommendations for enhancing access for primary care patients to rehabilitation in low-resource contexts, it does not determine whether these recommendations would lead to the desired outcome. Thus, more nuanced research is needed to assess the readiness, feasibility and impact of implementing these recommendations on health outcomes in these specific contexts.

The study involved a few selected PHC facilities and districts in one province in each country. The findings cannot be generalised to other districts, provinces or low-resource contexts. Quantitative research would be useful in obtaining a broader understanding of these recommendations in the countries. Finally, while the qualitative descriptive approach enabled us to explore the perspectives of both patients and PCPs, the participants were not involved in the analysis and interpretation of the data. Future research could employ more participatory research approaches that utilise high-level engagement with patients to provide additional unique insights.^59^

## Conclusion

This study provides practical context-specific strategies for ensuring that primary care patients in South Africa and Zimbabwe could have better access to quality rehabilitation. From the perspectives of PCPs and patients, key areas for improvement include providing rehabilitation services closer to home, improving patient awareness of rehabilitation, improving PCPs’ knowledge and basic skills in rehabilitation, improving communication among primary care stakeholders and advocating for rehabilitation inclusion at policy and leadership levels. Our findings underscore the urgent need to integrate rehabilitation into PHC to address the growing demand for these services amid epidemiological transitions and resource constraints. Future research can consider the impact of these recommendations on the quality and accessibility of rehabilitation services available to primary care patients. Primary care providers, rehabilitation service planners and policymakers can use these findings as a roadmap as they move towards strengthening rehabilitation in PHC.
